# Mass spectrometry-based analysis of glycoproteins and its clinical applications in cancer biomarker discovery

**DOI:** 10.1186/1559-0275-11-14

**Published:** 2014-04-10

**Authors:** Huan Liu, Ningbo Zhang, Debin Wan, Meng Cui, Zhiqiang Liu, Shuying Liu

**Affiliations:** 1 Changchun Center of Mass Spectrometry, Changchun Institute of Applied Chemistry, Chinese Academy of Sciences, 5625 Renmin Street, Changchun 130022, P. R. China; 2 University of the Chinese Academy of Sciences, Beijing 100039, P. R. China

**Keywords:** Mass spectrometry, Glycoproteins, Cancer biomarker

## Abstract

Glycosylation is one of the most important posttranslational modifications of proteins and plays essential roles in various biological processes. Aberration in the glycan moieties of glycoproteins is associated with many diseases. It is especially critical to develop the rapid and sensitive methods for analysis of aberrant glycoproteins associated with diseases. Mass spectrometry (MS) has become a powerful tool for glycoprotein analysis. Especially, tandem mass spectrometry can provide highly informative fragments for structural identification of glycoproteins. This review provides an overview of the development of MS technologies and their applications in identification of abnormal glycoproteins and glycans in human serum to screen cancer biomarkers in recent years.

## Introduction

Glycoproteins consist of oligosaccharide chains covalently attached to polypeptide side-chains and play essential roles in a wide range of biological processes, as well as in disease genesis and progression [1-3]. The two most common glycosylation forms of protein include O-glycosylation on Ser/Thr residues and N-glycosylation on Asn residue with the consensus sequence Asn-Xaa-Ser/Thr (Xaa = any amino acid except proline (Pro)) [1]. Abnormal glycosylation of proteins has been implicated to play a key role in cancerous progression, such as breast cancer, ovarian cancer, and prostate cancer and so on [2,4,5]. It is very important to elucidate the structures of glycoproteins. However, there are challenges in the analysis of glycoproteins in biological samples as follows:

• The native target glycoproteins are often low-abundant;

• It is hard to perform the analysis of glycoproteins by simultaneous profiling of protein sequences, glycan moieties and glycosylation sites;

• The complexity and heterogeneity of glycan structures increase the complexity of structural analysis of glycoproteins;

• The sensitive and quantitative methods are urgently needed to detect specific glycoform of glycoprotein associated with disease progression.

Over the years, mass spectrometry (MS) has become a powerful tool to determine glycoproteins due to its outstanding 4S features (sensitivity, stoichiometry, specificity and speed) [6-12], which facilitates the identification of the glycoproteins, glycosylation sites and structures of glycans. For example, based on ‘top-down’ mass spectrometry-based approach, O-glycoprotein sequence and O-glycosylation sites can be obtained by MALDI in-source decay (ISD) TOF/TOF-MS [13]. The peptide sequences, glycosylation sites and glycan moieties of N-glycopeptides were profiled by combination of high resolution/accurate mass and multiple dissociation techniques (CID, ETD, HCD) on the LTQ Orbitrap XL mass spectrometer [14]. The isomeric glycans were successfully differentiated by ion mobility spectrometry (IMS) MS [15] and nano-LC-Chip/TOF-MS [16]. The specific glycoform of α2,6-linked tri-sialylated triantennary glycan of haptoglobin associated with human lung cancer was identified and quantified using MALDI-Q/TOF-MS/MS, MALDI-TOF/TOF and nanoESI-orbitrap mass spectrometers [17].

However, due to the challenges mentioned above, purification, enrichment and fractionation approaches are of the essence prior to MS analysis. Several comprehensive reviews about sample pretreatment of glycoproteins have been published in recent years [18-20], so we will not discuss them here. The current review focuses on the development of MS technologies and their application in the analysis of abnormal glycoproteins in biological samples to screen cancer biomarkers in recent years.

### Mass spectrometry

There are two general MS-based strategies for glycoprotein analysis. One is the “top-down” MS-based strategy that the intact glycoproteins are directly subjected to MS and tandem MS analysis to provide the protein sequencing ladders and in situ localization of complex glycans without extensive separation or digestion [13,21,22]. This approach just needs minimal sample preparation and it has been applied to analysis of bacterial flagella glycoproteins [22]. FT-ICR-MS and TOF-MS are often employed for "top-down" analysis with high resolution and mass accuracy. Until now, this strategy has been limited to small glycoproteins, and has not been used for clinical glycoproteomic analysis due to the complex MS spectrum interpretation and sample separation. The other is the “bottom-up” MS-based strategy that is the most widely applied to analysis of glycoproteins. It includes two common analytical approaches. One approach is to the release of glycans from the glycoproteins by chemical or enzymatic methods, and then the carbohydrates and proteins are purified and analyzed, respectively. For N-linked glycoproteins, typical N-glycanase (PNGase) enzymes are used, such as PNGase F [23,24]. For O-linked glycoproteins, the release of glycans is often accomplished by chemical methods (such as β-elimination) [25]. Derivatization of carbohydrates is often necessary to improve the MS analysis [25-27]. The deglycosylated proteins are digested by endoprotease, such as trypsin, and then determined by MS. The structures of glycans and sequences of proteins can be obtained. Unfortunately, the information of binding sites of carbohydrates on proteins could be lost. The other approach is to directly digest glycoproteins with endoprotease, and then digested glycopeptides are characterized by mass spectrometry [28]. The glycosylation sites can be determined. These two approaches are complementary in the identification of glycoproteins.

### Ion sources

Electrospray ionization (ESI) and matrix-assisted laser desorption ionization (MALDI) are two soft ionization technologies which are widely applied to the analysis of glycoproteins and glycans. Usually, multiply charged ions (M + nH)^n+^ of glycoproteins are generated by ESI-MS, which makes it possible to detect the intact glycoproteins and simultaneously increases the challenge of the interpretation of complicated data. Nano-electrospray ionization (nano-ESI) shows high sensitivity and low sample consumption due to decreased initial droplet sizes. Derivatization of glycoproteins is commonly used to improve ionization efficiency [25]. ESI is very compatible with liquid chromatography (LC) to realize on-line LC-MS analysis. By two-dimensional nano-LC-nano-ESI-MS/MS, multiple N-linked glycoproteins were identified [29]. Compared with ESI, glycoproteins often carry singly charged species or low-charge ions during vacuum MALDI ionization, which simplifies the data analysis. Vacuum MALDI has the capacities of higher throughput and more tolerance for contaminants than ESI. But the loss of labile monomers of glycan moiety or glycoprotein is the major drawback of vacuum MALDI [25]. Permethylation of glycans can stabilize the labile glycosidic bonds.

### Mass analyzers

There are five common mass analyzers for analysis of glycoproteins/glycans in clinical samples: ion trap (IT), quadrupole, time-of-flight (TOF), orbitrap and Fourier transform ion cyclotron resonance (FT-ICR) mass spectrometers [6-8,10,12,30]. These analyzers are summarized in Table 1. Among all mass analyzers, FT-ICR-MS possesses highest mass accuracy, ultrahigh resolution and high cost. It is used for the characterization of N- or O-glycans [23,25,30,31] and glycoproteins [32]. For orbitrap instrument, although its resolution (up to 150,000) can not match that of FT-ICR-MS, the operation of orbitrap is relatively simple and convenient without the need of superconducting magnets [14]. It is very compatible with nano-LC for identification of glycoproteins in cancer samples [33].

**Table 1:** Types of mass analyzers

**Mass analyzer**	**Sampling**	**Separation**	**Typical resolution**	**Mass accuracy**	**Tandem mass spectrometry**	
**Quadrupole**	Continuous ion beam	Trajectory stability	2,000	100 ppm	Triple quadrupoles: CID (precursor ion scan, product ion scan, neutral loss)	Low energy collision
**Ion trap**	Continuous ion beam	Frequency	4,000	100 ppm	CID MS^n^, ETD, ECD	Low energy collision
**TOF**	Pulsed ion beam	Flight time	10 000	10 ppm for reflection TOF	TOF/TOF: CID	High energy collision
					Q-TOF: CID	Low energy collision
**Orbitrap**	Pulsed ion beam	Frequency	100,000	<5 ppm	CID, HCD, ETD	Low energy collision
**FT-ICR**	Pulsed ion beam	Frequency	10^5^-10^6^	<5 ppm	SORI-CID, ECD, IRMPD, EDD	Low energy collision

Generally, hybrid analyzer instruments are extensively applied to identify glycoproteins, because such hybrid species could combine the merits of different analyzers. For example, MALDI TOF/TOF MS instrument is widespread applied to identify glycosylation sites and glycan structures and quantify the changes of abundance of glycans [34,35]. Linear ion trap quadrupole (LTQ) instrument and hybrid Q/TOF instrument equipped with online LC separation system are used for clinical glycoprotein analysis [29,36-38]. Combined with a linear ion trap, orbitrap has been already employed to identify glycoprotein with high resolution and mass accuracy [14], which provides rapid and accurate tandem MS analysis (CID, ETD, HCD) of complex compounds.

### Tandem mass spectrometry

Tandem mass spectrometry has become the powerful tool for identification of the peptide/protein sequences, glycosylation sites and glycan structures (i.e. composition, sequence and linkage), such as collision-induced dissociation (CID), electron capture dissociation (ECD), electron transfer dissociation (ETD), higher-energy collision dissociation (HCD, formerly higher-energy C-trap dissociation), infrared multiphoton dissociation (IRMPD) and electron detachment dissociation (EDD) [6,9,12,30,39].

CID technique is the most common dissociation technique. According to the collision energy, it can be divided into the low-energy CID (a few volts to a few hundred volts) and high-energy CID (in the kilovolt range). The different diagnostic fragment ions can be produced. In general, at relatively low CID energies (typically 10^−1^–1 eV collisions in IT and FT-ICR analyzers and 10–10^2^ eV collisions in tandem quadrupole (TQ) and Q-TOF) [9], glycosidic bonds of glycopeptides are often broken to produce B-/Y-type fragment ions in the positive mode for the analysis of the glycan structures [9]. (The nomenclature of carbohydrate fragmentations was developed by Domon and Costello [40]). Usually, the collision energies for the cleavages of glycosidic bonds are much lower than those for cleavages of peptide bonds, so few peptide sequence information can be provided. The oxonium-type ions generated by CID, IRMPD and HCD are often used to locate glycopeptides. In the negative-ion CID, the complementary structural information of glycopeptides can be also obtained [41,42]. Various types of fragment ions of N-glycan, such as B/Y-type ions, C-type glycosidic fragment ions, A-type cross-ring fragment ions as well as D-type and E-type ions can be identified by CID in negative mode and the rich information about linkages and positional isomers can also be provided [42]. Some fragmentations of peptide backbones can be also observed. By high-energy CID (typically 10^3^–10^4^ eV in TOF/TOF instrument [9]), the fragmentation of peptide bonds was observed in spectra as well as the fragmentation of glycoside bonds. Cross-ring fragment ions of the glycan moiety can be generated to provide the linkage and branching information of glycan residues [34]. In addition, combination of CID with high resolution mass spectrometers will provide rapid and sensitive identification of glycopeptides [43]. However, CID technique has limitation for the determination of glycosylation sites due to the labile nature of N-glycan attached to peptide chain.

ETD and ECD are typical electron transfer and capture dissociation techniques. ECD can be performed in FT-ICR MS instrument and ion-trap MS instrument. ETD can be implemented in ion trap and ion trap-orbitrap MS instrument. Generally, by ETD, the cleavages of peptide bonds of glycopeptides are produced to generate c/z-type fragment ions to provide the peptide sequences. During the fragmentation, the glycan moieties remain attached to peptides, which provide glycosylation sites [14,44,45]. For oligosaccharides, various types of cross-ring cleavage ions can be generated by ETD to clarify the different linkage types and branching patterns of the representative milk sugar samples [46]. By ECD, alkali, alkaline earth, and transition metals were utilized to generate multiple positively charged ions for oligosaccharides [47]. Cross-ring cleavage is the dominant fragmentation pathway in ECD.

HCD is performed in an orbitrap mass spectrometer. The collision energy of HCD is higher than that of CID in the linear ion trap, and the resolution of fragment ions are very high [44]. The cleavages of both peptide bonds and glycosidic bonds are observed, which could provide the information of peptide sequences and glycan structures [14,44,45].

IRMPD fragmentation of glycopeptides in FT-ICR instrument is dominated by the cleavages of glycosidic bonds and peptide backbonds [48,49], which depend on some factors, such as the charge state, charge carrier, glycan composition, and peptide composition [49]. For example, in IRMPD, singly protonated glycopeptide ions containing a basic amino acid residue almost exclusively cleaved peptide backbone, but doubly protonated glycopeptides resulted in glycosidic bond cleavage. Sodiated glycopeptides generated more glycan fragment ions by IRMPD than protonated glycopeptides. The most abundant fragment ion by Y1 cleavage of glycan had been observed in IRMPD spectra. These fragments are important for the determination of the N-glycan structure types and glycosylation sites.

In FT-ICR mass spectrometer, EDD resulted in more extensive glycosidic cleavages and cross-ring cleavages for sialylated oligosaccharides and provided complementary structural information with IRMPD [48].

The combination of different fragmentation techniques plays an important role in comprehensive characterization of glycoproteins. Ye, H-P et al. reported the application of online LC-MS full scan with alternating CID, ETD and HCD scans for characterization of model glycoproteins [14]. 23 glycoforms derived from all 5 corresponding glycopeptides by glycoprotein digestion were detected. Glycan structures were elucidated in CID. Glycosylation sites were clearly localized by ETD fragmentation data. Peptide analysis was accomplished using HCD. ETD and CID provided complementary fragment information which comprehensively elaborated the complex intact glycopeptides. HCD provided supplemental information for both glycan sequences and peptide sequences.

In addition, differentiation of isomeric carbohydrates is much challenged by mass spectrometry alone. One approach is to apply multiple-stage tandem mass spectrometry (MS^n^) to find the specific fragment ions of different isomers. The other approach is to employ ion mobility spectrometry (IMS) to separate the isomers according to the difference of their collisional cross sections [15]. Meanwhile, high-field asymmetric waveform ion mobility spectrometry (FAIMS) is also applied to carbohydrate analysis [50].

### LC-MS technique

Due to complexity and heterogeneity of glycan structures on glycoproteins, it is the challenges for their structural identification and quantitation in biological samples. Generally, high performance separation techniques are necessary for analysis of closely related structures of glycan moieties prior to MS analysis. Compared with conventional LC, nano-LC has higher sensitivity and higher efficiency in the separation of glycopeptides or glycans [16,51]. However, there are still some limitations, including the high back pressure and low reproducibility [52]. Combined with the microfluidic chip packed with porous graphitized carbon (PGC), nano-LC-MS was used for N-glycan profiling with high stability and repeatability, and N-glycan isomers were differentiated [16,42,53].

### Clinical applications in cancer biomarker discovery

Biomarkers are of the essence to monitor cancer or disease progression; likewise quantitative methods are indispensable to screen potential markers in human samples for clinical applications. Generally, two relative quantitation strategies are used to quantify glycans and glycoproteins for discovering glycan biomarkers and glycoprotein biomarkers. The label-free quantitative strategies are widely used by profiling the changes of abundances of altered glycans or glycoproteins using MS by comparing a cancer sample to a control sample [54,55]. Some label quantitative strategies are also utilized, such as isotopic labeling [2,27], metabolic labeling [5] and iTRAQ labeling [29,33,56,57]. MALDI MS is often employed for quantitation of the abundances of N- or O-glycans with (or without) permethylation [25,31,54]. ESI-MS coupled with nano-LC is widely used for glycans and glycopeptides released from glycoproteins [2,25,27,55]. These detailed quantitative strategies were summarized and evaluated in previous reviews [58,59] and are not discussed here.

### Released glycans

For potential glycan biomarker discovery, comparative changes of glycans between cancer samples and controls are identified and quantified. Glycans are released from glycoproteins, purified, labeled, and subjected to MS analysis. It is sensitive to discover altered glycans as promising markers at glycosylation levels.

#### N-glycans

N-glycans generally were enzymatically released by N-glycosidase F (PNGase F) from serum glycoproteins, purified and (or) permethylated, and subjected to MS analysis.

Goldman et al. quantitatively assessed 83 N-glycans with permethylation originating from serum samples including 73 HCC cases, 77 age- and gender-matched cancer-free controls, and 52 patients with chronic liver disease using MALDI-TOF MS through the altered abundance of N-glycans in HCC samples in comparison to that of cancer-free controls [54]. The abundances of 57 N-glycans were significantly altered in HCC patients compared with controls. With 90% prediction sensitivity, the combination of three identified N-glycans (m/z 2,472.9, 3,241.9, and 4,052.2) was sufficient for the detection of HCC in a population with high rates of hepatitis C viral infection. The changes of three identified N-glycans were more suitable as markers for clinical utility than serum α-fetoprotein used currently as clinical marker. This method is effectively used to quantify N-glycans with high sensitivity and high specificity for a large clinical sample analysis.

Kamiyama et al. developed a glycoblotting method using BlotGlycoH to purify N-glycans from HCC preoperative blood samples from 369 patients and 26 normal controls using MALDI-TOF MS analysis [60]. The novel analytical method, glycoblotting, has been evidenced to be far more rapid and accurate for N-glycan analysis. A total of 67 N-glycans had higher relative areas in the HCC cases compared with the normal controls. G3560 (m/z 3560.295) and G2890 (m/z 2890.052) N-glycans using univariate and multivariate analysis were found to be strongly correlated with tumor number, size, and vascular invasion, so they were proved to be more accurate than the well-known biomarkers for predicting HCC tumor malignancy including microscopic portal vein invasion, and the patient survival and disease-free survival rates. Compared with Goldman's study, this strategy is rapid and simple for N-glycan analysis from HCC without permethylation treatment but obtains more clinical information.

De Leoz et al. examined N-linked glycan variations during breast cancer progression using transplantable breast tumor mouse model and breast cancer patients and controls [31]. Released N-glycans were purified and fractionated by solid phase extraction (SPE) using a graphitized carbon cartridge, and quantitatively profiled by MALDI FT-ICR MS using an internal standard without permethylation treatment. The elevation of a high-mannose type glycan containing nine mannoses, Man9 (m/z 1,905.630), was observed in both mouse and human sera in the presence of breast cancer. Elevated Man9 was found to be an incompletion of the glycosylation process.

Balog et al. developed a high separation power method to quantify N-glycans labeled with 2-aminobenzoic acid from 13 colorectal cancer tumor tissues and control colon tissues using the combination of hydrophilic interaction liquid chromatography (HILIC-HPLC) and MALDI-TOF-MS [61]. The result showed that a bisecting GlcNAc was decreased and sulfated glycans, paucimannosidic glycans, and glycans containing a sialylated Lewis type epitope were shown to be increased in tumor tissues. HILIC-HPLC has an excellent separation power for some small N-glycans.

It is very challenged to distinguish isomeric N-glycans associated with cancers. Fortunately, IMS-MS is developed for the isomeric glycan analysis in clinical applications [15,62]. Isailovic et al. described a high-throughput IMS-MS analysis of N-linked glycans from human serum including 22 healthy control patients and 20 individuals with cirrhosis of the liver and 19 individuals with liver cancer [15]. IMS-MS provided information about glycan conformational and isomeric composition. Statistical analysis for glycan data suggested that aberrations in isomer distributions were probably indicative of some disease states.

Hua et al. developed the nano-LC-Chip/TOF-MS method to identify and quantify isomeric native N-glycans from human serum of prostate cancer patients with high reproducibility [16]. A microfluidic chip packed with graphitized carbon was used to N-glycan separation. Over 300 N-glycan species with 100 N-glycan compositions were identified and the abundance changes of specific type N-glycans were determined between poor prognoses and good prognoses.

Site-specific analysis of protein glycosylation is also important for disease progression. For example, α1-3/4 fucosylation at Asn 241 of β-haptoglobin (β-Hp) glycan was a new biomarker for colon cancer [63]. α 2,6-linked tri-sialylated triantennary glycan of haptoglobin from the serum was a potential glyco-biomarker for the diagnosis of human lung cancer [17]. The glycoforms at specific positions on glycoprotein were detected by LC separation in conjunction with MS^n^[17,63,64].

Wang et al. developed a LC-LTQ-CID/ETD-MS method to identify the site-specific glycosylation of haptoglobin from lung cancer patient plasma using porous layer open tubular (PLOT) LC column [64]. Ultranarrow PLOT LC column (2.5 m × 10 μm i.d.) with low-level sample consumption provided high sensitivity for optimal separation of glycopeptides. Full-scan MS, CID-MS^2^ and ETD-MS^2^ of LTQ MS provided glycopeptide structure information including glycoforms and glycosylation sites. A total of 26 glycoforms on 3 tryptic N-glycopeptides from haptoglobin were identified and quantified. This strategy was highly sensitive for analysis of N-linked protein glycosylation heterogeneity and low abundant glycopeptides at about 100 amol level.

#### O-glycans

The O-glycans are usually released from O-glycoproteins by reductive β-elimination method because no enzymes are suitable to release the O-glycans. Wada et al. employed various methodologies to assess the O-glycans from three samples of IgA1 isolated from the serum of patients with multiple myeloma (coded NUD, Sap-II, and VDS) [25]. Three main MS strategies were used to identify and quantify reductively eliminated O-glycans. The first strategy was that permethylated glycans were analyzed by MALDI-MS in positive ion mode, and sequenced by MALDI and/or ESI-MS/MS. In the second strategy, native glycans were separated by graphitized carbon on-line LC, analyzed by ESI-MS in negative ion mode, and sequenced by ESI-MS/MS. In the third strategy, mixtures of native glycans without on-line LC purification were analyzed by MALDI-FT-ICR-MS in negative or positive ion mode. The quantitative data were obtained from MALDI-MS and ESI-MS by measuring peak heights and peak areas, respectively. Some monosaccharides were not detected in the on-line LC experiments probably because of matrix peak disturbance in MALDI and early elution together with impurities in on-line LC-MS. Three MS strategies were preeminent to analyze O-glycans from IgA1 samples. The first two-strategies were reliable to quantitatively profile O-glycans, but the third one was irresponsible by reason of equivocal data. These semi-quantitative MS methods are preeminent for profile of O-glycans from serum and facilitate O-glycan marker discovery.

### Glycoproteins

The glycoproteins are digested by trypsin to generate nonglycosylation peptides and glycopeptides. Glycopeptides, deglycosylation peptides or nonglycosylation peptides derived from targeted glycoproteins in cancer samples can be analyzed by MS. This strategy is excellent to detect altered glycoproteins at glycoprotein level.

#### N-glycoproteins

For N-glycoprotein analysis, N-glycoproteins were enriched using hydrazide chemistry and lectin capture to remove nonglycoproteins, digested by trypsin and PNGase F treatment. Generated glycopeptides or deglycosylation peptides or nonglycosylation peptides were identified and quantified by LC-ESI-MS/MS.

Tian et al. first developed a high throughput method for quantitative analysis of altered expression of sialoglycoproteins in breast cancer [2]. Modified solid phase extraction of glycopeptides (SPEG), in which glycopeptides were treated with sialidase, specifically enriched sialoglycopeptides. Glycopeptides isolated by modified and original SPEG were released by PNGase F from human serum, labeled by d0, d4 (four deuterium), or d4C4 (four deuterium and four ^13^C)-succinic anhydride, and identified and quantified by LC-MS/MS (LTQ ion trap MS). Changes in expression of sialoglycoproteins and total glycoproteins associated with breast cancer were identified. Versican, one of the sialoglycoproteins, was most sialylated and elevated in breast cancer using lectin and Western blot analysis. Immunohistochemistry study revealed that epithelial expression of versican had significant relation to lymph node metastasis and pathological stages. The modified SPEG specifically capturing sialoglycopeptides is simple and straight-forward for high throughput analysis. This method is very good to identify altered expression of sialoglycoproteins associated with cancers.

Li et al. also used SPEG method to isolate N-glycoproteins in 8 cases of bronchoalveolar lavage (BAL) fluid, 8 lung adenocarcinoma tissues and 8 tumor-matched normal lung tissues [33]. Deglycosylation peptides from isolated N-glycoproteins were labeled by iTRAQ and analyzed by LC-orbitrap-HCD-MS/MS. The result showed that 25 glycoproteins were at least 2-fold difference between cancer and benign BAL. The levels of Napsin A in cancer BAL were further verified using an ELISA assay. The combination of SPEG, stabilized iTRAQ labeling and high resolution MS is very helpful for analysis of low abundance glycoproteins in biological samples.

Wu et al. used lectin array method to identify and confirm differentially expressed fucosylated glycoproteins in serum of patients with different stage ovarian cancer [36]. Fucosylated glycoproteins extracted using LCA and UEAI lectins were labeled with isobaric chemical tags labeling. Deglycosylation peptides from fucosylated glycoproteins were analyzed by LC-MS/MS (LTQ). Five differentially expressed glycoproteins were found in the serum of ovarian cancer patients compared to benign diseases and confirmed by lectin-ELISA and ELISA assay. Compared to CA125 alone, the combination of CBG, SAP and CA125 showed high performance for distinguishing stage III ovarian cancer from benign diseases. The similar method was applied to identify and confirm differentially expressed sialoglycoproteins in the serum of patients with ovarian cancer [4]. Sambucus nigra (SNA) lectin was used to extract sialylated glycoproteins. Compared to CA125 alone (AUC = 0.811), the combination of CLUS and LRG1 (AUC = 0.837) showed high performance for distinguishing stage III ovarian cancer from benign diseases. By two methods above, independent sample sets were used to verify the ability of fucosylated and sialylated glycoproteins as candidate markers to detect patients with ovarian cancer.

Ahn et al. used multiple reaction monitoring (MRM) technique to quantify glycopeptides in both total plasma and its aleuria aurantia lectin (AAL)-captured fraction of each HCC and hepatitis B virus (HBV) sample [65]. AAL was utilized to capture fucose-specific glycoforms. Fucosylated glycoform levels of target glycoproteins from HCC and HBV plasmas were confirmed to be different. Additionally, A1AT and FETUA as fucosylated biomarker candidates mainly increased in fucosylation levels on these target glycoproteins between HCC and control groups. The high specific and sensitive MRM-based analysis was a successful approach to quantify aberrant protein glycosylation in cancer plasma samples.

Chen et al. found aberrant change in IgG_1_ Fc-glycosylation in human serum associated with age, sex, female sex hormones and cancers in large clinical samples [66,67]. IgG was separated with SDS-PAGE from serum and digested by trypsin. Generated glycopeptides were enriched with SPE and analyzed by MALDI-FTICR MS (9.4 T hybrid Qh-FTICR). The marked increase in IgG_1_ Fc-agalactosylation and decrease in galactosylation were observed in lung cancer patients compared to that in healthy controls [66]. In addition, diagnostic ability of IgG_1_ Fc-glycosylation was both sex and age dependent. This study is useful in clinical application associated with IgG_1_ Fc-glycosylation in human lung cancer.

Ahn et al. investigated aberrantly glycosylation of tissue inhibitor of metalloproteinase 1 (TIMP1) in colorectal cancer patients using MALDI-FTICR-MS with a stable isotope internal standard [32]. Phytohemagglutinin-L4(L-PHA) lectin was used to extract β-1,6-N-acetylglucosamine moiety of N-linked glycan on glycoprotein. It was also found that aberrant glycoforms of TIMP1 were about 5 times higher in colorectal cancer serum than that in the noncancerous serum. In this method, MALDI-FTICR-MS shows ultrahigh-resolution (Rs > 400, 000) and high mass accuracy (Δ < 0.5 ppm). However, MALDI-FTICR-MS is unsuitable for analyzing complex mixture due to their closely related glycoforms which cannot be differentiated in spectra.

Chen et al. developed a high throughput label-free quantitative method to analyze glycoprotein abundances in human serum associated with HCC [55]. Nonglycosylated peptides derived from captured glycoproteins were analyzed by LC-MS/MS (LTQ linear ion trap MS). 38 glycoproteins were found with concentration changes between HCC and normal samples, including α-fetoprotein which is the only clinical marker for HCC diagnosis. Abundance changes of three glycoproteins (galectin-3 binding protein, insulin-like growth factor binding protein 3 and thrombospondin 1) were obtained as potential markers for the development of HCC. This quantitative method of nonglycosylated peptides from capturing glycoproteins simplified the process of sample treatment and MS data analysis without need of analysis of N-glycosites. Due to simple sample treatment, this method could be used for the other cancer sample analysis.

#### O-glycoproteins

The profile of O-glycopeptides using MS technologies facilitates the analysis of altered O-glycoproteins in serum for clinical research. Wada et al. developed different methods for O-glycosylation profile for IgA1 from the serum of patients with multiple myeloma. MS methods to analyze O-glycopeptides were assessed [25]. Two main strategies based MS, i.e. MALDI-MS in linear TOF mode and on-line LC-ESI-MS, were used to analyze cysteine-alkylated tryptic hinge O-glycopeptides isolated from solution digests. Data obtained from most of these laboratories were remarkably consistent despite the use of a variety of sample handling procedures and MS instruments. However, there were some shortcomings of these methods. In the MALDI mass spectra, sialylated glycopeptides had the low efficiency of the ionization in the positive and negative ion mode and sialyl residues were lost on profile of oligosaccharides or glycopeptides. Jacalin lectin chromatography was used to isolate the glycopeptides. However, the result was insufficient for a comprehensive MS study due to the volume (2 ml) of jacalin was too great to efficiently recover the glycopeptides from the lectin column for smaller scale analysis.

## Conclusions

Although there is no universal method for comprehensive identification of glycoproteins, mass spectrometry has the great advantages in structural analysis of glycoproteins. Mass spectrometry-based strategies are extensively used to detect the altered glycan and glycoprotein expression in human cancer samples to find cancer biomarkers. At present, bottom-up MS-based strategy is dominated in glycoprotein analysis while top-down MS-based approach still has some limitations. However, the characterization of glycoproteins is not always comprehensive that either glycans or glycosylation peptides are detected without intact glycoprotein analysis. With the improvement of MS instruments, we believe top-down approach will become a convenient, sensitive and rapid way to directly analyze the glycoprotein without time-consuming digestion and separation in the future. There is another approach called “middle-down” approach in proteomics that proteins are digested to larger peptides for MS analysis. This method could be complementary to bottom-up and top-down approaches for the identification of glycoproteins. In addition, the improvements of purification, enrichment and fractionation will be great helpful for the characterization and quantitation of glycoproteins. MS-based strategies provide the valuable insights to better understand the cancer development and progression.

## Competing interests

The authors declare that they have no competing interests.

## Authors’ contributions

All authors discussed and contributed to the content of the text. HL drafted the manuscript. All authors read and approved the final manuscript.
